# Etymologia: Poliomyelitis

**DOI:** 10.3201/eid2508.ET2508

**Published:** 2019-08

**Authors:** Ronnie Henry

**Keywords:** poliomyelitis, polio, poliovirus, viruses, infantile paralysis, vaccines, Ruma, Michael Underwood, Karl Landsteiner, Erwin Popper, Thomas Weller, Frederick Robbins, Jonas Salk, Albert Sabin, *Suggested citation for this article*: Etymologia: Poliomyelitis. Emerg Infect Dis. 2019 Aug [*date cited*]. https://doi.org/10.3201/eid2508.ET2508

## Poliomyelitis [pō'-lē-ō-mī-ə-lī-ʹtəs]

From the Greek *polios* (“gray”) + *myelos* (“marrow”), poliomyelitis may have plagued humanity since antiquity. The funerary stele of the Egyptian priest Ruma (circa 1400 bce) shows a shortened, withered leg, in what is believed to be one of the earliest depictions of polio. The first clinical description was in 1789 by Michael Underwood. Karl Landsteiner and Erwin Popper identified poliovirus in 1908, and 40 years later John Enders, Thomas Weller, and Frederick Robbins were able to grow poliovirus in tissue culture cells, work for which they received the Nobel Prize in Medicine or Physiology in 1954. This breakthrough facilitated vaccine research, and the first inactivated polio vaccine, developed by Jonas Salk and his team, was licensed in 1955. Six years later, Albert Sabin and his team developed a live, attenuated oral polio vaccine (Figure).

**Figure:**
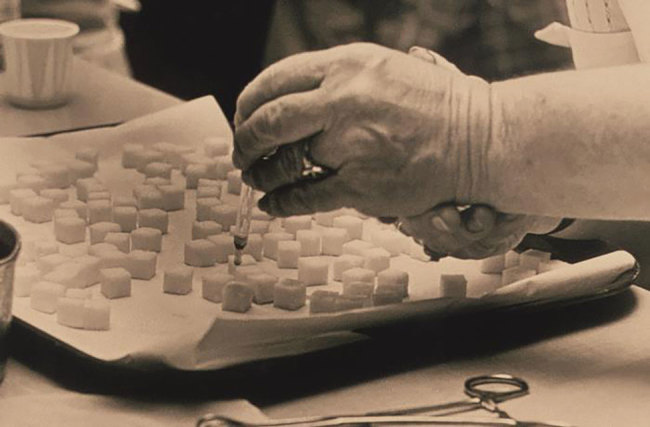
This historic 1975 photograph shows a laboratory technician preparing doses of polio vaccine by placing a liquid droplet of the vaccine on each of these sugar cubes, which would subsequently be ingested orally by each recipient. Photo: Public Health Image Library, Centers for Disease Control and Prevention, 1975

Because broad immunization campaigns made progress toward regional polio elimination in the Americas, in 1988 the World Health Assembly declared a goal of global polio eradication. Through a partnership between Rotary International, the World Health Organization, the United Nations Children’s Fund, the Centers for Disease Control and Prevention, and the Bill & Melinda Gates Foundation, the Global Polio Eradication Initiative has achieved a 99.9% decrease in the global incidence of polio. Today, wild poliovirus transmission occurs in only Afghanistan and Pakistan, and 4 of the 6 World Health Organization regions have formally declared the elimination of the indigenous wild poliovirus. Of the 3 types of poliovirus, type 2 wild poliovirus was declared eradicated globally in 2015, and type 3 wild poliovirus has not been detected since 2012. With only 33 cases globally from type 1 wild poliovirus in 2018, the task remains to eliminate polio in its last niches.
